# A Translation-Independent Directed Evolution Strategy to Engineer Aminoacyl-tRNA Synthetases

**DOI:** 10.1021/acscentsci.3c01557

**Published:** 2024-05-20

**Authors:** Chintan Soni, Noam Prywes, Matthew Hall, Malavika A. Nair, David F. Savage, Alanna Schepartz, Abhishek Chatterjee

**Affiliations:** † Department of Chemistry, Boston College, Chestnut Hill, Massachusetts 02467, United States; ‡ Innovative Genomics Institute, University of California, Berkeley, California 94720, United States; § Howard Hughes Medical Institute, University of California, Berkeley, California 94720, United States; ∥ Department of Biology, Boston College, Chestnut Hill, Massachusetts 02467, United States; ⊥ Department of Molecular and Cellular Biology, University of California, Berkeley, California 94720 United States; # Department of Chemistry, University of California, Berkeley, California 94720, United States; 7 California Institute for Quantitative Biosciences, University of California, Berkeley, California 94720, United States; 8 Chan Zuckerberg Biohub, San Francisco, California 94158, United States; 9 ARC Institute, Palo Alto, California 94304, United States

## Abstract

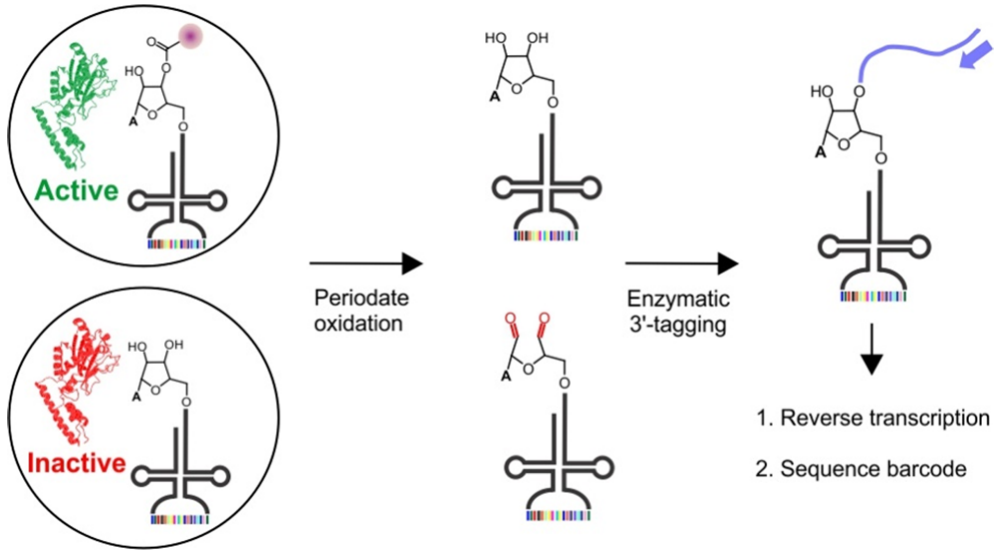

Using directed evolution, aminoacyl-tRNA synthetases (aaRSs) have been engineered to incorporate numerous noncanonical amino acids (ncAAs). Until now, the selection of such novel aaRS mutants has relied on the expression of a selectable reporter protein. However, such translation-dependent selections are incompatible with exotic monomers that are suboptimal substrates for the ribosome. A two-step solution is needed to overcome this limitation: (A) engineering an aaRS to charge the exotic monomer, without ribosomal translation; (B) subsequent engineering of the ribosome to accept the resulting acyl-tRNA for translation. Here, we report a platform for aaRS engineering that directly selects tRNA-acylation without ribosomal translation (START). In START, each distinct aaRS mutant is correlated to a cognate tRNA containing a unique sequence barcode. Acylation by an active aaRS mutant protects the corresponding barcode-containing tRNAs from oxidative treatment designed to damage the 3′-terminus of the uncharged tRNAs. Sequencing of these surviving barcode-containing tRNAs is then used to reveal the identity of the aaRS mutants that acylated the correlated tRNA sequences. The efficacy of START was demonstrated by identifying novel mutants of the *Methanomethylophilus alvus* pyrrolysyl-tRNA synthetase from a naïve library that enables incorporation of ncAAs into proteins in living cells.

## Abstract 2

A novel approach to engineer aminoacyl-tRNA synthetases is reported that does not rely on translational readout. It will enable the selection of mutants for charging monomers that are poor substrates for the wild-type ribosome.

## Introduction

Over the last two decades, the genetic code of cells in various domains of life has been expanded to include hundreds of noncanonical amino acids (ncAAs) with diverse chemical structures.^1−4^ This expansion has been accomplished by developing engineered aminoacyl-tRNA synthetase/tRNA pairs that selectively incorporate various ncAAs in response to a unique codon (e.g., a repurposed nonsense codon).^1−5^ The ability to site-specifically incorporate enabling ncAAs into proteins using this technology has unlocked powerful new ways to probe and manipulate protein function for both basic science and biotechnology applications.^1−3,6,7^

Despite such exciting progress, this technology has been largely restricted to the incorporation of simple l-α-amino acids and, to a lesser extent, some α-hydroxy acids.^8−12^ Pioneering early work using *in vitro* translation systems has demonstrated that the ribosome has the potential to accept a much wider variety of monomers for translation such as d-α-amino acids,^13^ β- and γ-amino acids,^14−17^ long-chain amino acids,^18^ aminobenzoic acid,^19^ α-aminoxy and α-hydrazino acids,^20^ α-thio acids,^21^ and others.^22−24^ The remarkable tolerance of the ribosome for such noncanonical monomers and further expansion of its noncanonical substrate scope through engineering^25−28^ offer a possible path for repurposing mRNA-templated ribosomal translation to synthesize nonpeptide polymers.

**Figure 1:**
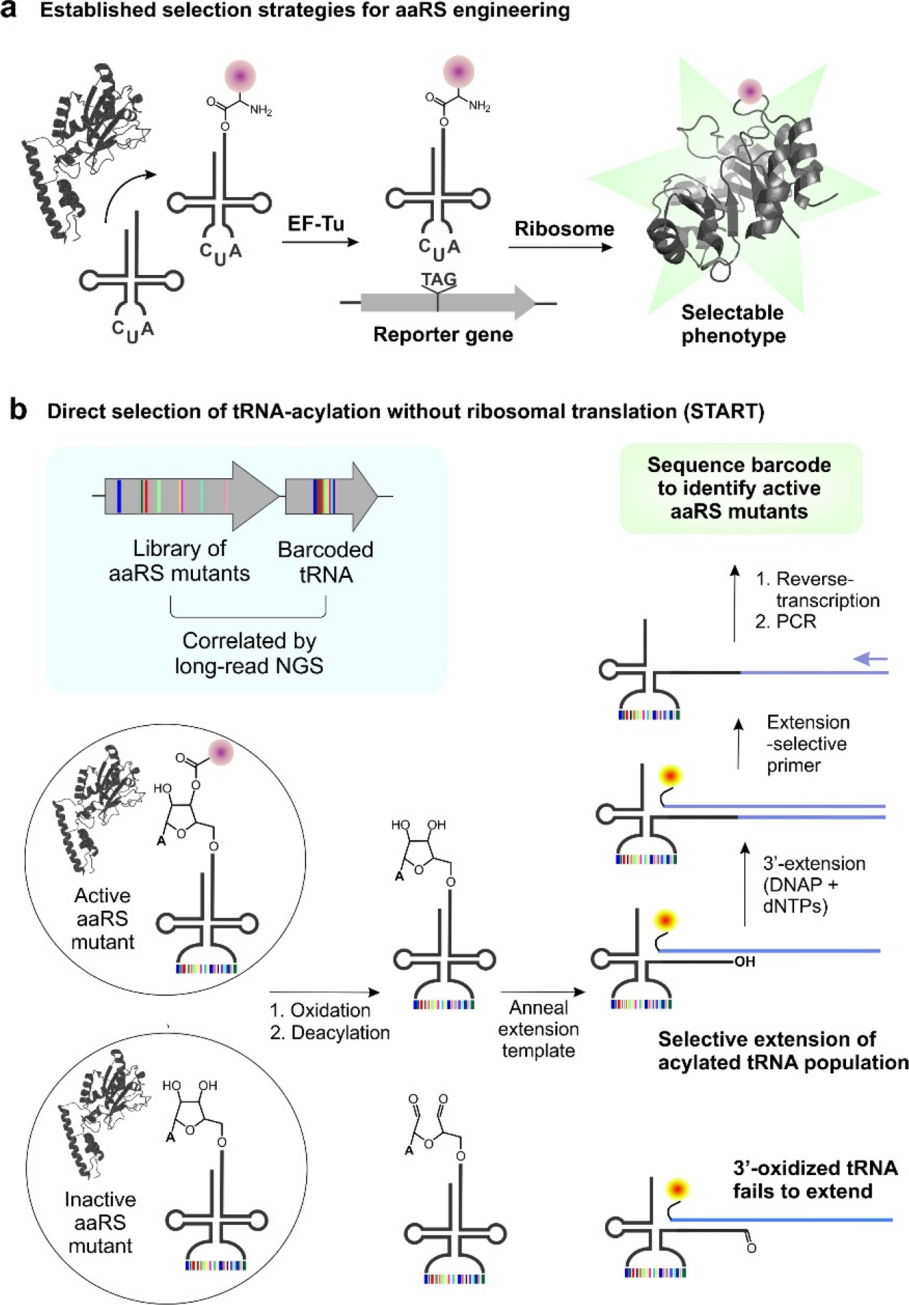
Directed evolution strategies for engineering the substrate specificity of aaRSs. (a) Established selection strategies depend on ribosomal translation to connect the activity of aaRS to the expression of a reporter protein with a selectable phenotype. This strategy is incompatible with noncanonical monomers that are poor substrates for the ribosome. (b) In START, members in an aaRS mutant library are individually correlated to a sequence barcode located within the cognate tRNA. Successful charging by a mutant aaRS protects its cognate barcode-containing tRNAs from a periodate-mediated oxidation, enabling their subsequent enrichment via templated 3′-extension and RT-PCR.

Although incorporation of structurally divergent monomers into peptides has been broadly explored *in vitro*, examples of achieving the same in living cells remain scarce. The key bottleneck limiting progress on this front is the lack of engineered aminoacyl-tRNA synthetases (aaRSs) capable of efficiently charging such monomers. In the handful of examples, where such exotic monomers were incorporated into proteins in *E. coli*, such as β^3^-*p*-bromo-homophenylalanine^26^ and a β^2^-hydroxy acid,^29^ tRNA acylation relied on promiscuous recognition that certain wild-type aaRSs fortuitously exhibited for these substrates. The ability to systematically engineer aaRSs to charge a wider variety of noncanonical monomers with high fidelity and efficiency is needed to overcome this limitation. However, established directed evolution strategies for altering the substrate specificity of aaRSs rely on the ribosomal translation of reporter proteins with a selectable phenotype (such as antibiotic resistance or fluorescence; Figure 1a).^30−39^ Unfortunately, such translation-dependent selection schemes are incompatible with noncanonical monomers that are poor substrates for the ribosome. Although the ribosome could be engineered to accept novel monomers, such efforts would require access to engineered aaRSs capable of generating monomer-charged tRNAs. Engineering of both the aaRS and the ribosome at the same time is challenging, as it would require co-localization of two rare mutants from each library in the same cell, which is associated with low statistical odds. Consequently, a two-step solution is needed, where the aaRS is first engineered to charge the noncanonical monomer without relying on translational readout, which can then be used for ribosome engineering.

Here we report a general approach for engineering aaRSs that involves direct selection of tRNA acylation without ribosomal translation (START; Figure 1b). Our approach was inspired by an established strategy to enrich acylated tRNA pools from living cells, where the 2′,3′-dihydroxy functionality at the 3′-terminus of uncharged tRNAs is selectively oxidized using periodate.^40−47^ The acylated tRNAs are protected from this damage and can be subsequently tagged with a unique oligonucleotide sequence at its intact 3′-terminus, allowing their subsequent identification through selective reverse-transcription and PCR (RT-PCR).^42−47^ Although this strategy is effective for enriching and characterizing acylated tRNA sequences, it does not reveal the identity of the aaRS responsible for their acylation. To do so, it is essential to connect the identity of each aaRS variant to its cognate tRNA sequence. In START, we achieve this connection by introducing a sequence barcode into a permissive site within the tRNA. Using *Methanomethylophilus alvus* pyrrolysyl-tRNA (tRNA^MaPyl^) as the model system, we demonstrate the feasibility of inserting a sequence barcode in the anticodon loop with a limited impact on tRNA expression and charging. Next, the START selection scheme was optimized to allow robust enrichment of an acylated barcode-containing tRNA^MaPyl^ from a mixed population that also contained an uncharged tRNA^MaPyl^ with a distinct barcode. Then, we created an active site library of *M. alvus* pyrrolysyl-tRNA synthetase (MaPylRS), where each unique mutant is encoded by associated tRNA^MaPyl^ barcodes, and subjected this library to the START selection scheme to identify MaPylRS mutants capable of charging different ncAAs. Finally, we show that the START selection scheme is compatible with non-α-amino acid monomers. START represents the first translation-independent directed evolution platform for aaRS engineering, which will be invaluable for developing incorporation systems for unique noncanonical monomers.

## Results and Discussion

### A Chemical Strategy to Select for Charged tRNAs

A translation-independent directed evolution strategy would ideally allow the selection of active aaRS mutants simply based on their ability to acylate a cognate tRNA with a desired noncanonical monomer. Upon aaRS-catalyzed acylation of a tRNA on its 3′-terminal ribose, it becomes protected from oxidation by sodium periodate, which selectively oxidizes vicinal diol groups. This method has long been leveraged to characterize the charging status of tRNAs in cells.^40−47^ Recently, this strategy has been combined with modern enrichment and sequencing methods to perform such analyses in a more high-throughput manner.^42,43,45−47^ After the selective periodate-mediated oxidation of the uncharged tRNAs and the deacylation of the charged tRNAs using mildly alkaline conditions, a unique oligonucleotide sequence can be introduced onto the intact 3′-terminus of the acylated population either through ligation^43,45,46^ or by polymerase-mediated extension of the 3′-terminus using a DNA template that is hybridized onto the tRNA sequence.^44^ The installed oligonucleotide sequence can be subsequently used to selectively reverse-transcribe and PCR amplify the charged tRNA sequences.

**Figure 2:**
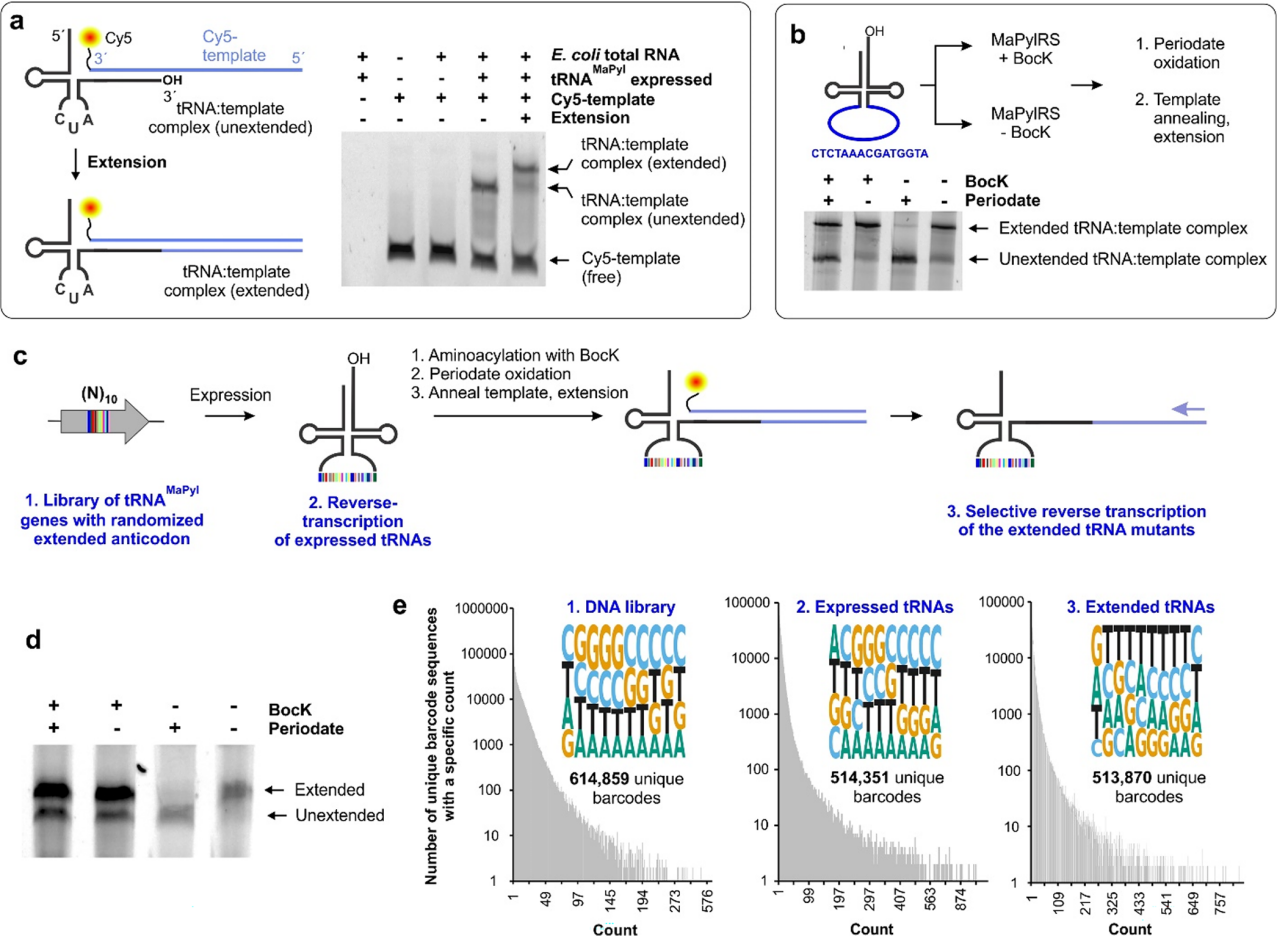
The anticodon loop of tRNA^MaPyl^ can be expanded to include a sequence barcode. (a) 3′-extension method used to selectively tag tRNA^MaPyl^ with an intact 3′-end, using a 3′-Cy5-labeled DNA template. Native PAGE followed by fluorescence imaging shows that the designed Cy5-DNA template selectively hybridizes with tRNA^MaPyl^ and enables the formation of the extension product. (b) A tRNA^MaPyl^ mutant with a severely expanded anticodon loop can be effectively charged by MaPylRS, as revealed by the tRNA-extension assay. In the absence of tRNA acylation, periodate treatment prevents the formation of the extension product, but when coexpressed with MaPylRS and a cognate ncAA substrate (BocK), robust formation of extension product was observed, indicating successful acylation of the extended tRNA^MaPyl^. (c) Scheme for characterizing the expression and potential aminoacylation of the members of the tRNA^MaPyl^ barcode library. (d) A large majority of the barcode-containing tRNA^MaPyl^ library members are successfully acylated in the presence of MaPylRS and BocK, as revealed by protection from periodate oxidation in the tRNA-extension assay. (e) Illumina sequencing of the barcode-containing tRNA^MaPyl^ library DNA (left), after it is expressed in *E. coli* (middle), and members that survive periodate treatment when coexpressed with MaPylRS in the presence of BocK (right) reveal similar distribution and composition. These analyses indicate that a majority of the barcode-containing tRNA^MaPyl^ sequences are successfully expressed and acylated by MaPylRS.

The methods described above for the selective enrichment of acylated tRNAs have the potential to serve as the foundation for a translation-independent selection strategy for tRNA acylation. To explore this possibility, we focused on the *M. alvus*-derived pyrrolysyl-tRNA synthetase (MaPylRS)/tRNA^MaPyl^ pair as a model system.^48^ The pyrrolysyl pair represents the leading platform for expanding the genetic code with both ncAAs and non-α-amino acid monomers.^1−3,9,24,29,49^ We designed a 3′-fluorophore-labeled DNA template (Figure S1) that will hybridize with tRNA^MaPyl^ and allow the extension of its 3′-terminus by DNA polymerase (Figure 2a). This template selectively hybridized with tRNA^MaPyl^ expressed in *E. coli*, as observed by nondenaturing PAGE followed by fluorescence imaging; no such complex was found when total RNA from *E. coli* not expressing the tRNA^MaPyl^ was used instead (Figure 2a). Incubation of this DNA:tRNA complex with the Klenow fragment of *E. coli* DNA polymerase (lacks 3′ → 5′ exonuclease activity) and dNTPs led to successful extension of the 3′-terminus, as shown by an upward shift in the PAGE analysis. Treatment of tRNA^MaPyl^ with sodium periodate prevented the appearance of this extension product, consistent with the oxidation of its free 3′-terminus (Figure S2). However, when tRNA^MaPyl^ was coexpressed with MaPylRS in the presence of a known ncAA substrate (N^ε^-Boc-l-lysine; BocK), it was protected from periodate-mediate damage and successfully yielded the extension product (Figure S2). These results confirm that the tRNA-extension strategy can be used to selectively introduce an oligonucleotide tag at the 3′-terminus of the acylated tRNA^MaPyl^ population, allowing for their subsequent enrichment by RT-PCR.

### Expanding the Anticodon Loop of tRNA^MaPyl^ to Insert a Sequence Barcode

To use this strategy for evolving MaPylRS, its sequence information must be encoded within tRNA^MaPyl^ such that the identity of variants responsible for tRNA acylation can be retrieved by sequencing the acylated tRNA^MaPyl^ pool following their enrichment. We envisioned achieving this encoding by introducing a sequence barcode within a permissive site of tRNA^MaPyl^. The anticodon loop of tRNA^MaPyl^ is a promising location to introduce the barcode, given pyrrolysyl synthetases do not interact with the anticodon region.^50−55^ Indeed, the anticodons of pyrrolysyl-tRNAs have been altered to enable suppression of other nonsense codons, as well as four-base codons, indicating significant plasticity.^53−56^

To explore whether the anticodon loop of tRNA^MaPyl^ can tolerate more dramatic expansions, we generated a variant where the native sequence in the anticodon loop was replaced with a random 15-nucleotide sequence (Figure 2b). The resulting expanded tRNA^MaPyl^ mutant was coexpressed in *E. coli* with MaPylRS in either the presence or absence of a cognate ncAA substrate (BocK). Total RNA isolated from these cells was subjected to a tRNA-extension assay with or without periodate treatment (Figure 2b). The successful expression of the expanded tRNA^MaPyl^ mutant was confirmed by the appearance of the tRNA:DNA hybrid band. When incubated with DNA polymerase and dNTPs, expanded tRNA^MaPyl^ successfully yielded the extension product in the absence of periodate treatment. MaPylRS was found to efficiently acylate the expanded tRNA^MaPyl^ variant with BocK, as the formation of the extension product was largely insensitive to periodate when the ncAA was included in the growth medium, but almost fully disappeared in its absence (Figure 2b).

Although this result was encouraging, we sought to further confirm that the anticodon loop of tRNA^MaPyl^ would tolerate a broad variety of sequences without significantly compromising its expression or aminoacylation by MaPylRS, which is essential to accessing sufficient barcode diversity for encoding a MaPylRS mutant library. To this end, we replaced the anticodon loop of tRNA^MaPyl^ with a sequence of 10 fully randomized nucleotides to create a library of roughly 1 × 10^6^ mutants (Figure 2c). Illumina sequencing (approximately 5 × 10^6^ usable reads) of the resulting plasmid DNA encoding this tRNA library revealed the presence of >600,000 unique barcodes (Figure 2e). Each nucleotide was well-represented at each position of the barcode library (Figure 2e). Additionally, >90% of the barcodes in the library were found to have an abundance that is within a single standard deviation from the average (∼8.5 counts/barcode), indicating reasonably good diversity and distribution of the barcode sequences. To evaluate the expression patterns of the barcode-containing tRNA^MaPyl^ genes, this plasmid library was transformed into *E. coli*, and the expressed tRNA^MaPyl^ sequences were amplified by RT-PCR and analyzed by Illumina sequencing. Over 500,000 unique sequences were identified in the expressed tRNA^MaPyl^ barcode library, and the representation of each nucleotide in the barcode was comparable to the DNA library (Figure 2e; Figure S3). These observations confirm that a majority of the barcode-containing tRNA^MaPyl^ sequences can be expressed in *E. coli*.

Finally, to explore if MaPylRS can charge barcode-containing tRNA^MaPyl^ sequences, the library was coexpressed with MaPylRS in *E. coli* in the presence of BocK. The resulting barcode-containing tRNA^MaPyl^ pool was oxidized by periodate to damage the uncharged population, and the acylated sequences were subjected to tRNA extension using a 3′-Cy5-labeled DNA template (Figure 2c). Analysis of this extension reaction by PAGE followed by fluorescence imaging (Figure 2d) showed that most of the tRNA^MaPyl^ pool was protected from periodate oxidation in the presence of BocK (but not when the ncAA was absent), indicating that a large majority of barcode-containing tRNA^MaPyl^ sequences are efficiently charged by MaPylRS. This finding was further confirmed by amplifying the acylated barcode-containing tRNA^MaPyl^ population by RT-PCR and analyzing them by Illumina sequencing. Greater than 500,000 unique barcode sequences were identified in the acylated fraction, and their composition and distribution were similar to the library at the DNA and expressed tRNA level (Figure 2e). Together, these experiments show that expanding the tRNA^MaPyl^ anticodon loop is a viable approach for incorporating a diverse sequence barcode that does not drastically disrupt the expression or charging by MaPylRS.

### Optimization of START to Enrich Charged, Barcode-Containing tRNA^MaPyl^ Sequences from a Mixed Population

**Figure 3:**
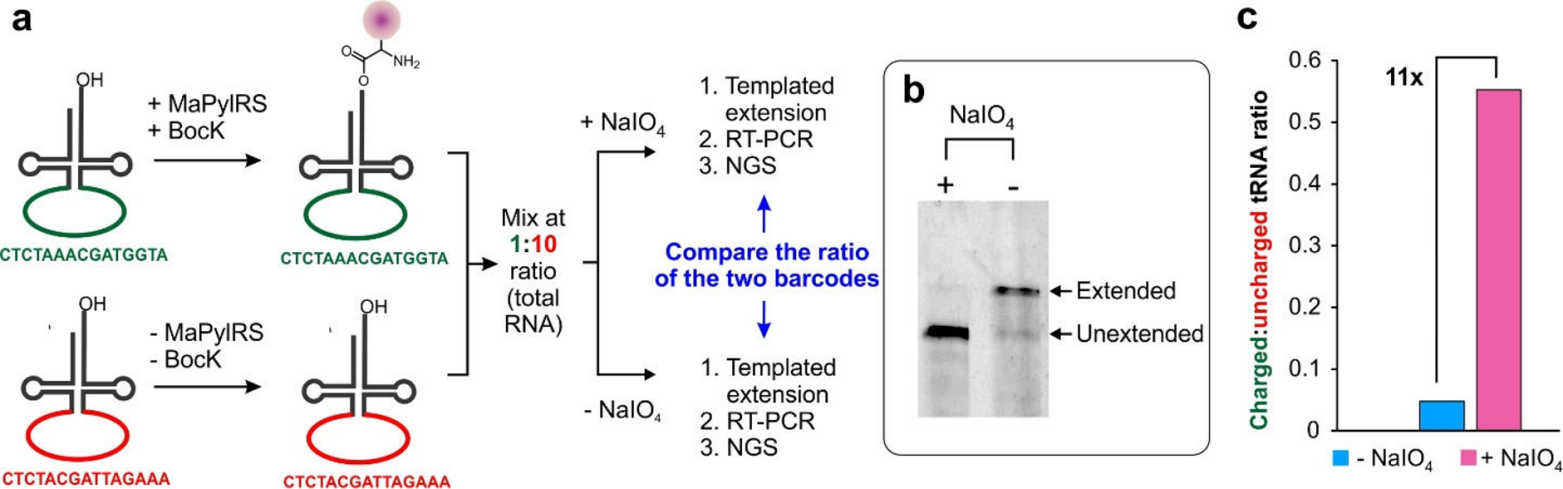
START enables the enrichment of an acylated barcode-containing tRNA^MaPyl^ from a defined mixed population. (a) Scheme of the experiment showing two distinct barcode-containing tRNA^MaPyl^ are separately expressed, where one is charged with BocK and the other is not. A defined mixture of these two populations is subjected to the START scheme, and their relative abundance is quantitatively measured in the presence and absence of the periodate-mediated selection using amplicon sequencing. (b) PAGE followed by fluorescence imaging of the tRNA extension reaction of this mixed population in the presence or absence of periodate oxidation. (c) Amplicon sequencing reveals that the acylated tRNA^MaPyl^ barcodes are enriched approximately 11-fold upon periodate selection.

The basic steps of the START scheme involve periodate oxidation to damage the uncharged tRNAs, selective DNA-templated extension of the charged tRNAs to introduce a unique sequence at the 3′-terminus, followed by its use to amplify these sequences by RT-PCR. To improve the enrichment of the desired population, we employed a gel purification step to isolate the extended DNA:RNA hybrid from the unextended counterparts based on its different mobility on nondenaturing PAGE. We explored different lengths of the DNA template to alter the extended product size and optimize its separation from other species (Figure S4). Next, for evaluating the performance of this selection scheme, we created two standard plasmids to represent an “active” or “inactive” member in the library (Figure 3a). Each plasmid encoded a distinct barcode-containing tRNA^MaPyl^ sequence, but one of these also encoded wild-type MaPylRS, which will acylate the corresponding tRNA^MaPyl^ in the presence of BocK and represent an active library member. The other plasmid lacked MaPylRS and would represent an inactive library member. These plasmids were separately transformed into *E. coli*, and using the aforementioned tRNA-extension assay, we confirmed that the tRNA^MaPyl^ variant coexpressed with MaPylRS was protected from oxidative damage in the presence of BocK, whereas its counterpart lacking MaPylRS was not (Figure S5). In order to quantify the degree of enrichment afforded by our strategy, the mock inactive and active tRNA^MaPyl^ populations were mixed in 10:1 ratio, and the composition of these mixed population was characterized by amplicon sequencing (∼50,000 reads) before and after subjecting it to the START scheme (Figure 3a,b). The tRNA^MaPyl^ variant coexpressed with MaPylRS was found to undergo roughly an 11-fold enrichment upon selection (Figure 3c), suggesting that this strategy should allow differentiation between barcode-containing tRNA^MaPyl^ variants associated with active or inactive MaPylRS mutants.

### Identification of ncAA-Selective MaPylRS Mutants Using START

**Figure 4:**
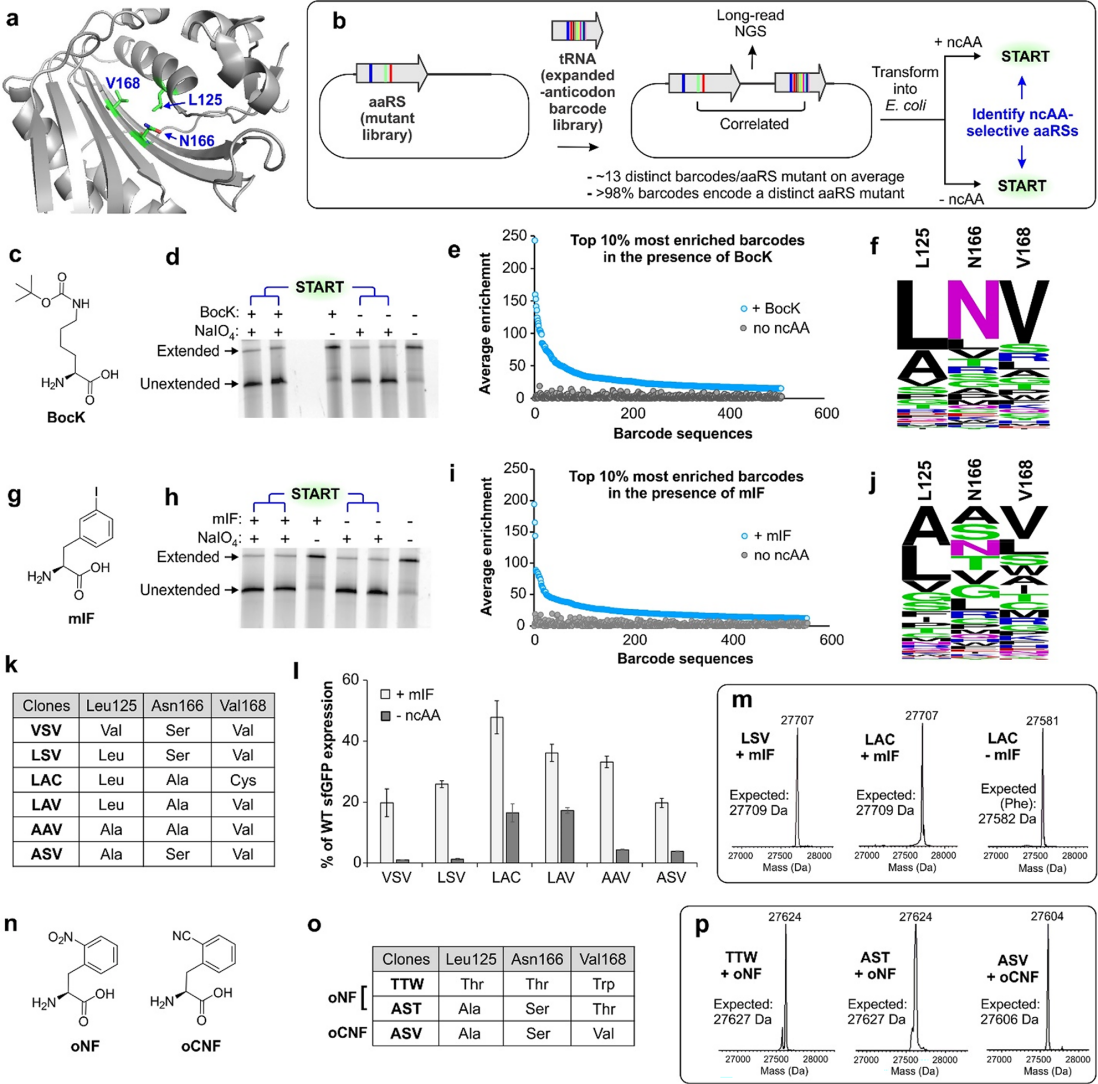
Selection of ncAA-selective MaPylRS mutants using START. (a) The active site of MaPylRS, where the three key residues randomized to generate the library are highlighted. (b) Scheme for generating and selecting the MaPylRS library, where each mutant is correlated to distinct barcode-containing tRNA^MaPyl^ sequences. (c) Structure of BocK. (d) PAGE followed by fluorescence imaging of the tRNA-extension reaction on the library in the presence and absence of BocK. (e) Top 10% most enriched sequence barcodes that show at least 2-fold higher enrichment in the presence of BocK (relative to no BocK). Enrichment of each barcode in the presence (blue) or the absence (gray) of BocK is shown. (f) WebLogo analyses of the residues observed in the active sites of the MaPylRS mutants correlated to these top 10% barcodes reveal the wild-type MaPylRS sequence as the most prevalent. (g) The structure of mIF. (h) PAGE followed by fluorescence imaging of the tRNA-extension reaction on the library in the presence and absence of mIF. (i) Top 10% most enriched sequence barcodes that show at least 2-fold higher enrichment in the presence of mIF (relative to no mIF). Enrichment of each barcode in the presence (blue) or the absence (gray) of mIF is shown. (j) WebLogo analyses of the residues observed in the active sites of the MaPylRS mutants correlated to these top 10% barcodes reveal that the prevalent signature is distinct from that of the wild-type MaPylRS. (k) Selected clones for further characterizations, based on their average enrichment levels, the number of associated barcodes that show enrichment, and similarity to the consensus sequence of the most enriched sequences (panel j). (l) The mIF-charging activity of each MaPylRS mutant, upon coexpression with tRNA_CUA_^MaPyl^, measured as the expression of a sfGFP-151-TAG reporter in the presence/absence of mIF and normalized to the expression of a wild-type sfGFP reporter. The characteristic fluorescence of the reporter was measured in cells resuspended in PBS. (m) ESI-MS analysis of purified sfGFP-151-TAG reporter for LSV and LAC mutants in the presence of mIF shows masses consistent with the incorporation of mIF at the TAG codon. In the absence of mIF, the LAC mutant likely charges phenylalanine, as indicated by the mass of the isolated reporter protein. (n) The structures of oNF and oCNF. (o) MaPylRS clones selected using START (see Figures S8 and S9 in the SI for details) to be characterized for charging oNF and oCNF. (p) ESI-MS analysis of purified sfGFP-151-TAG reporters expressed using these mutants in the presence of the appropriate ncAA shows masses consistent with the incorporation of oNF or oCNF at the TAG codon.

Next, we explored if START can be used to identify MaPylRS mutants with altered substrate specificities from a naïve library. To this end, we simultaneously randomized three key residues (Leu125, Asn166, and Val168) around the substrate binding pocket of MaPylRS to NNK codons (Figure 4a), creating a library with a theoretical diversity of 32,768. Into the plasmid encoding the MaPylRS library, we further introduced our barcode-containing tRNA^MaPyl^ library containing an (N)_10_ randomized sequence at the anticodon loop (Figure 4b) using standard restriction cloning. The resulting library was covered using approximately 1.5 × 10^5^ transformants to ensure that (A) each barcode corresponds to a distinct MaPylRS mutant and (B) each MaPylRS is associated with multiple distinct barcodes. Since this library utilizes only ∼15% of all possible barcode sequences (∼10^6^), the chances of two different MaPylRS mutants receiving the same barcode are low. Additionally, as barcode diversity in the resulting library far exceeds the diversity of the MaPylRS library (roughly 50-fold), each distinct MaPylRS mutant should be associated with multiple unique barcodes, which would be beneficial to reduce the risk of false positives.

To characterize this library and correlate each unique MaPylRS mutant to the associated sequence barcodes, we used PacBio HiFi long-read DNA sequencing. From ∼1.2 × 10^5^ usable reads retrieved from the sequencing analysis, we confirmed the presence of >93% of all possible MaPylRS mutants. We also found that >91% of unique MaPylRS mutants were represented by more than one barcode, with the average being ∼13 barcodes/mutant (Figure S6a). Furthermore, approximately 93% of the barcodes were found to encode a single MaPylRS mutant (Figure S6b), which minimizes potential confusion when establishing genotype–phenotype connections.

Following characterization, the barcoded MaPylRS library was transformed to *E. coli* for subsequent selection. It is important to note that many MaPylRS active site mutants may charge one of the 20 canonical amino acids, which will protect the associated barcode-containing tRNAs from the periodate oxidation, generating false positives. Traditional aaRS engineering strategies typically employ a negative selection step to deplete such cross-reactive mutants.^1,30,31,33,34^ However, using the deep sequence coverage from NGS, it should be possible to characterize the enrichment pattern of nearly all mutants present in the MaPylRS library in response to the START scheme, and doing so in parallel in the presence and absence of a desired ncAA should help identify mutants that were selectively enriched in the presence of ncAA (Figure 4b). A similar approach was recently used for orthogonal tRNA evolution in mammalian cells.^57^

Using this strategy, the barcoded MaPylRS library was selected for its ability to charge two distinct ncAAs, BocK (Figure 4c–f) and *m*-iodo-l-phenylalanine (mIF) (Figure 4g–j), performed in duplicates. After selection, the surviving barcodes were amplified and characterized by Illumina sequencing. To identify barcodes associated with ncAA-selective MaPylRS mutants, we filtered the NGS data based on three key criteria: (A) it must be represented in the collection of barcodes obtained by long-read sequencing characterization of the original library, (B) it must show at least a 2-fold enrichment upon selection in the presence of ncAA, and (C) its relative enrichment in the presence of the ncAA must be at least 2-fold higher than in the absence. The top 10% (∼500) of the surviving barcodes were arranged in a decreasing order of average enrichment in the presence of the ncAA (Figure 4e and i) and were used to retrieve the sequences of the corresponding MaPylRS mutants. These sequences were used to generate a WebLogo sequence map that represents the relative abundance of the observed amino acid residues at each of the three randomized positions within this selected mutant pool. We were encouraged to find that the most enriched sequences for the BocK-selection matched with the wild-type MaPylRS (Figure 4f). This is expected since BocK is an excellent substrate for wild-type MaPylRS. In contrast, the most enriched mutants for the mIF selection had a distinct sequence signature (Figure 4j). We used the following criteria to select six distinct MaPylRS mutants (Figure 4k, Figure S7) from this pool of enriched sequences for individually assessing the ability to charge mIF: (A) having multiple associated barcodes that show enrichment, (B) high average enrichment observed across the correlated barcodes, and (C) similarity to the consensus sequence, i.e., the amino acid residues most frequently observed in each of the randomized positions in the most enriched MaPylRS mutants. These six MaPylRS mutants were coexpressed in *E. coli* with tRNA_CUA_^MaPyl^ and assessed for their ability to express sfGFP-151-TAG in the presence and absence of mIF (Figure 4l). Gratifyingly, all six mutants exhibited robust reporter expression in the presence of mIF (between 20% and 50% wild-type sfGFP). Although four mutants (those containing VSV, LSV, AAV, and ASV at positions L125, N166, and V168, respectively) exhibited low activity in the absence of mIF, LAC and LAV mutants maintained significant (albeit significantly less than +mIF) reporter expression. ESI-MS analysis of the purified reporter protein expressed using the LSV MaPylRS mutant confirmed incorporation of only mIF, when it was supplied in the growth medium (Figure 4m). Although the MaPylRS LAC mutant showed significant reporter expression in the absence of any ncAA, it was found to selectively charge mIF when it was present during expression (Figure 4m). This behavior has been previously documented for other engineered aaRSs as well and may result from its superior affinity for the ncAA relative to the canonical counterpart.^58−60^ We also isolated the reporter protein expressed using the MaPylRS LAC variant in the absence of mIF, and we observed a mass consistent with the incorporation of phenylalanine (Figure 4m).

Using the same MaPylRS library, we further applied the START protocol to identify MaPylRS mutants for charging ncAAs with two different ortho-substituted phenylalanine derivatives, *o*-nitro-l-phenylalanine (oNF) and *o*-cyano-l-phenylalanine (2-CNF). Following the same selection and analysis methods as described above, we identified TTW and AST as potential top candidates for charging oNF (Figure S8), and ASV was a potential top candidate for charging oCNF (Figure S9). All three MaPylRS mutants enabled robust expression of the sfGFP-151-TAG reporter in the presence of the appropriate ncAAs, and the ESI-MS analysis of the purified reporter protein confirmed selective incorporation of oNF (for TTW and AST) and oCNF (for ASV). These experiments further confirm that START can be used to identify novel aaRS mutants that accept noncanonical monomers as substrates from naïve libraries.

### START Is Compatible with Non-α-Amino Acid Monomers

**Figure 5:**
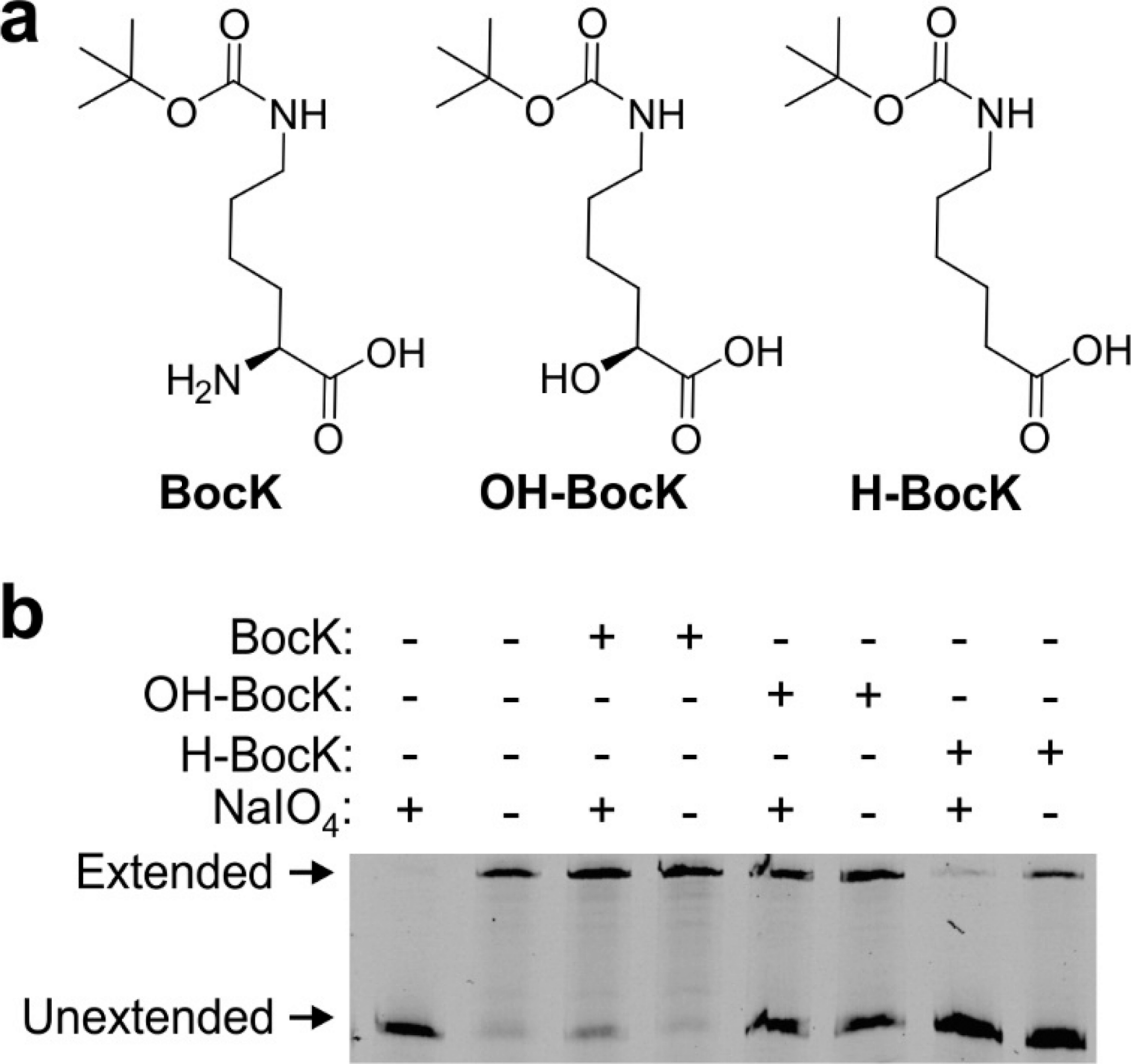
START scheme is compatible with non-α-amino acid monomers. (a) The structures of BocK, OH-BocK, and H-BocK. (b) Native PAGE followed by fluorescence imaging of tRNA-extension reaction performed on tRNA^MaPyl^ coexpressed with MaPylRS in the presence of BocK, OH-BocK, or H-BocK, or in the absence of any cognate substrate, and with or without periodate treatment. Successful formation of the extension product in the presence of each substrate, which is a known substrate for MaPylRS, confirms the compatibility of this selection with non-α-amino acid monomers.

Since the selection system reported here relies solely on the ability of an aaRS to acylate its cognate tRNA, it should be applicable to develop mutant aaRSs that charge noncanonical monomers whose structures diverge from those of the α-amino acids. It has been shown that the pyrrolysyl synthetases such as MaPylRS do not strongly recognize the α-amino group of the native substrate.^9^ This feature has enabled the use of this family of aaRSs to charge tRNA^Pyl^ with multiple substrate analogs in which the α-amino group is replaced with other functionalities, including α-hydroxyacids, desamino-acids, β^2^-hydroxyacids, etc., without further synthetase engineering.^10−12,24,26,29^ Using this demonstrated polyspecificity of native MaPylRS, we sought to explore whether the core selection system described herein is also compatible with non-α-amino acid monomers. *E. coli* cells coexpressing MaPylRS and tRNA^MaPyl^ were treated with BocK, OH-BocK, or H-BocK (Figure 5a) or in the absence of a potential substrate, and the resulting tRNA population was subjected to the tRNA-extension assay with or without a periodate treatment (Figure 5b). As expected, in the absence of any potential substrate, tRNA^MaPyl^ was uncharged and was susceptible to periodate oxidation, which prevented tRNA extension. The presence of BocK, OH-BocK, and H-BocK each facilitated the formation of the tRNA-extension product even with periodate treatment, confirming that the charging of this noncanonical monomer protects the tRNA from periodate oxidation and that this strategy may be potentially used to identify aaRS mutants selective for structurally diverse noncanonical monomers.

## Conclusion

There has been considerable interest in repurposing the mRNA-templated polypeptide synthesis by the ribosome to create novel sequence-defined polymers with noncanonical molecular architectures. However, translational incorporation of novel noncanonical monomers necessary for this purpose would require engineered variants of both aaRSs and the ribosome. Established aaRS engineering strategies are reliant on ribosomal translation and *vice versa*, thereby creating an interdependence that prevents systematic introduction of structurally novel noncanonical monomers into the genetic code. We offer a solution to this challenge by developing the first directed evolution strategy for engineering aaRSs, START, which does not rely on ribosomal translation. We show that tRNAs can be equipped with sequence barcodes, which can be correlated to individual mutants in large aaRS libraries through long-read, next-generation sequencing. Acylation of the correlated partner tRNA by a novel aaRS mutant protects it from periodate-mediated oxidation, allowing their subsequent enrichment. The identity of the corresponding aaRS mutant was then retrieved from the barcode sequence. The efficacy of this strategy was demonstrated by identifying novel mutants of MaPylRS selective for two ncAAs from a naïve library. The compatibility of this selection system with non-α-amino acids was further demonstrated by using the polyspecificity of MaPylRS for such substrates. It should be possible to extend the strategy described here to other aaRS/tRNA pairs. Although many aaRSs recognize the anticodon of its cognate tRNA as an identity element, which may complicate the introduction of a barcode in this region, it has been possible to engineer aaRS anticodon binding domains to be permissive for non-natural expanded anticodon sequences.^61,62^ Furthermore, such sequence barcodes can also be inserted into alternative locations within the tRNA to avoid aaRS identity elements. Indeed, it has been shown that aaRSs can effectively acylate significantly altered versions of their cognate tRNAs, including severely miniaturized versions.^63−66^

While this manuscript was under review, a similar strategy named tRNA display was reported for translation-independent engineering of *M. barkeri* PylRS (MbPylRS), wherein the cognate tRNA^Pyl^ was split at the anticodon region, and the MbPylRS mRNA was physically connected to the 3′-half of this split tRNA.^67^ While physically connecting the aaRS mRNA to a split tRNA offers certain advantages—such as the ability to perform multiple rounds of selection—splitting the tRNA while maintaining aaRS recognition is a complex undertaking that might be challenging to extend beyond the pyrrolysyl pair. By contrast, inserting a sequence barcode into a tRNA is considerably simpler, as discussed above, and could be more readily generalized. Nonetheless, the success of these two related translation-independent selection methods further corroborates the robustness of the underlying selection strategy, which offers an exciting opportunity to develop aaRS mutants to charge structurally divergent noncanonical monomers for the ribosomal synthesis of evolvable, sequence-defined polymers with unprecedented structure and function.

## Supporting Information Available

Experimental methods, nucleotide sequences, supplementary figures and tables (PDF)
